# Atrial fibrillation in patients hospitalized with acute myocardial infarction: analysis of the china acute myocardial infarction (CAMI) registry

**DOI:** 10.1186/s12872-016-0442-9

**Published:** 2017-01-04

**Authors:** Yan Dai, Jingang Yang, Zhan Gao, Haiyan Xu, Yi Sun, Yuan Wu, Xiaojin Gao, Wei Li, Yang Wang, Runlin Gao, Yuejin Yang, on behalf of the CAMI Registry study group

**Affiliations:** Department of Cardiology, Fuwai Hospital, National Center for Cardiovascular Diseases, Chinese Academy of Medical Science and Peking Union Medical College, 167 Beilishi Road, Beijing, 100037 People’s Republic of China

**Keywords:** Atrial fibrillation, Acute myocardial infarction, Hospital mortality, Anticoagulation treatment

## Abstract

**Background:**

The incidence, clinical outcomes and antithrombotic treatment spectrum of atrial fibrillation (AF) in patients hospitalized with acute myocardial infarction (AMI) have not been well studied in Chinese population.

**Methods:**

Twenty-six thousand five hundred ninety-two consecutive patients diagnosed with AMI were enrolled in CAMI registry from January 2013 to September 2014. After excluding 343 patients with uncertain AF status and 1,591 patients transferred out during hospitalization, 24,658 patients were finally included in this study and involved in analysis.

**Results:**

In the CAMI registry, 740 (3.0%) patients were recorded with AF prevalence during hospitalization. Higher-risk baseline clinical profile was observed in patients with AF. These patients were less likely to receive reperfusion/revascularization than those without AF. The in-hospital mortality (including death and treatment withdrawal) was significantly higher in patients with AF than that of without AF (25.2% vs. 7.2%, respectively; *p* < 0.01). The case of composite of adverse events was similar, which included death, treatment withdrawal, re-infarction, heart failure or stroke (42.1% vs. 16.0%, *p* <0.01). In multivariate logistic regression analysis, AF was an independent predictor for in-hospital mortality (odds ratio, 1.88; 95% confidence interval: 1.27–2.78) and the composite of adverse events (odds ratio, 2.11; 95% CI: 1.63–2.72). Only 5.1% of patients with AF were treated with warfarin, and 1.7% were treated with both warfarin and dual antiplatelet therapy.

**Conclusions:**

The analysis was based on the CAMI registry in China. The patients hospitalized for AMI who developed AF were at significantly higher risk for in-hospital mortality and other adverse events. However, the anticoagulants including warfarin have been largely underused post hospital discharge.

**Trial registration:**

Clinical Trial Registration: Identifier: NCT01874691.

## Background

Atrial fibrillation (AF) is a common complication of acute myocardial infarction (AMI). The reported incidence of AF was widely ranged from 2.3 to 21.0%, with an inconsistent relation to high mortality [1–12]. Although guidelines and consensus recommend a combination of warfarin and dual antiplatelet therapy (DAPT) and the duration was determined by hemorrhagic risk [13, 14], it was still complex to select an optimal antithrombotic regimen for patients with AF and AMI. Until now, this triple therapy has been largely underused in real-world clinical practice [15–17].

In China, AMI has become a major cause of emergency medical care, hospitalization and death over the past a few decades [18, 19]. The incidence, impact, and antithrombotic therapy of AF in AMI have not been correspondingly defined and demonstrated. Present analysis was aimed to study this subject with the data from China Acute Myocardial Infarction (CAMI) registry [20]. The data of patients with AMI were applied during January 2013 to September 2014. The baseline characteristics, treatment strategy, clinical data and outcomes were statistically analyzed and explored.

## Methods

### Study population

The design of the CAMI registry has been demonstrated in previous studies [20]. Briefly, this registry involved three levels of hospitals (representing typical Chinese governmental and administrative models) from all provinces and municipalities throughout mainland China (except for Hong Kong and Macau). Patients diagnosed with AMI were eligible for inclusion in CAMI registry, and were enrolled consecutively. Clinical data, treatments and outcomes were collected by local investigators and captured electronically with a fixed table, including a standardized set of variables and definitions, under a rigorous data quality control. A total of 108 hospitals have participated in the registry after its launch in January 2013 up to September 2014. This project was approved by the institutional review board central committee at Fuwai Hospital, National Center for Cardiovascular Diseases of China of China.

Inclusion and exclusion rules: the patients diagnosed with AMI in involved hospitals during January 2013 up to September 2014 were included. The patients were excluded if AF status was missing or they were transferred out during hospitalization. For the main analysis of in-hospital outcomes, the patients with truncated hospital stay because of outside transfer were also excluded. The presence of AF was documented by a standard 12-lead electrocardiogram or electrocardiogram monitoring during hospitalization.

### In-hospital outcomes

The primary outcome of this study was in-hospital mortality, which included death and treatment withdrawal (withdrawal from all medical therapy or premature hospital discharge). In China, many patients withdraw from treatment at terminal status, which could be attributed to the culture or financial affordability. Therefore, single in-hospital mortality without accounting for these patients could lead to an underestimate of actual in-hospital mortality rates. Other recorded in-hospital clinical events included: re-infarction, stroke, heart failure, a composite of adverse events (the combination of death, treatment withdrawal, re-infarction, heart failure or stroke), major bleeding (including an absolute hemoglobin decrease of 3 g/dL, intracranial hemorrhage, any red blood cell transfusion or a bleeding event requiring surgical repairing), and any reported bleeding. Detailed definitions of clinical events were previously demonstrated [20].

### Statistical analyses

The patient baseline characteristics, medical history, treatments, and complications were evaluated. Continuous variables are presented as median (interquartile range) and compared with Kruskal Wallis H test. Categorical variables were presented as counts and percentages, and were compared with chi-square or Fisher’s exact tests.

Logistic regression analysis was applied to evaluate the association between AF and in-hospital mortality or the composite of adverse events. The variables included in the multivariable model were either statistically significant on univariate analysis (*p* <0.05) or clinically critical, which were chosen by a stepwise method to minimize colinearity. Included covariates were: sex, age (>75 years), diabetes, hypertension, previous stroke, previous myocardial infarction, prior percutaneous coronary intervention (PCI)/coronary artery bypass graft (CABG), ST-segment elevation myocardial infarction (STEMI), serum creatinine, Global Registry of Acute Coronary Events (GRACE) score >140, CHA2DS2-VASc score >2, and reperfusion therapy. Crude and adjusted odds ratios (ORs) and corresponding 95% confidence intervals (CIs) were reported.

All comparisons were two-sided, with statistical significance defined as *p* less than 0.05. Statistical analysis was completed with SAS software, version 9.4.

## Results

**Fig. 1:**
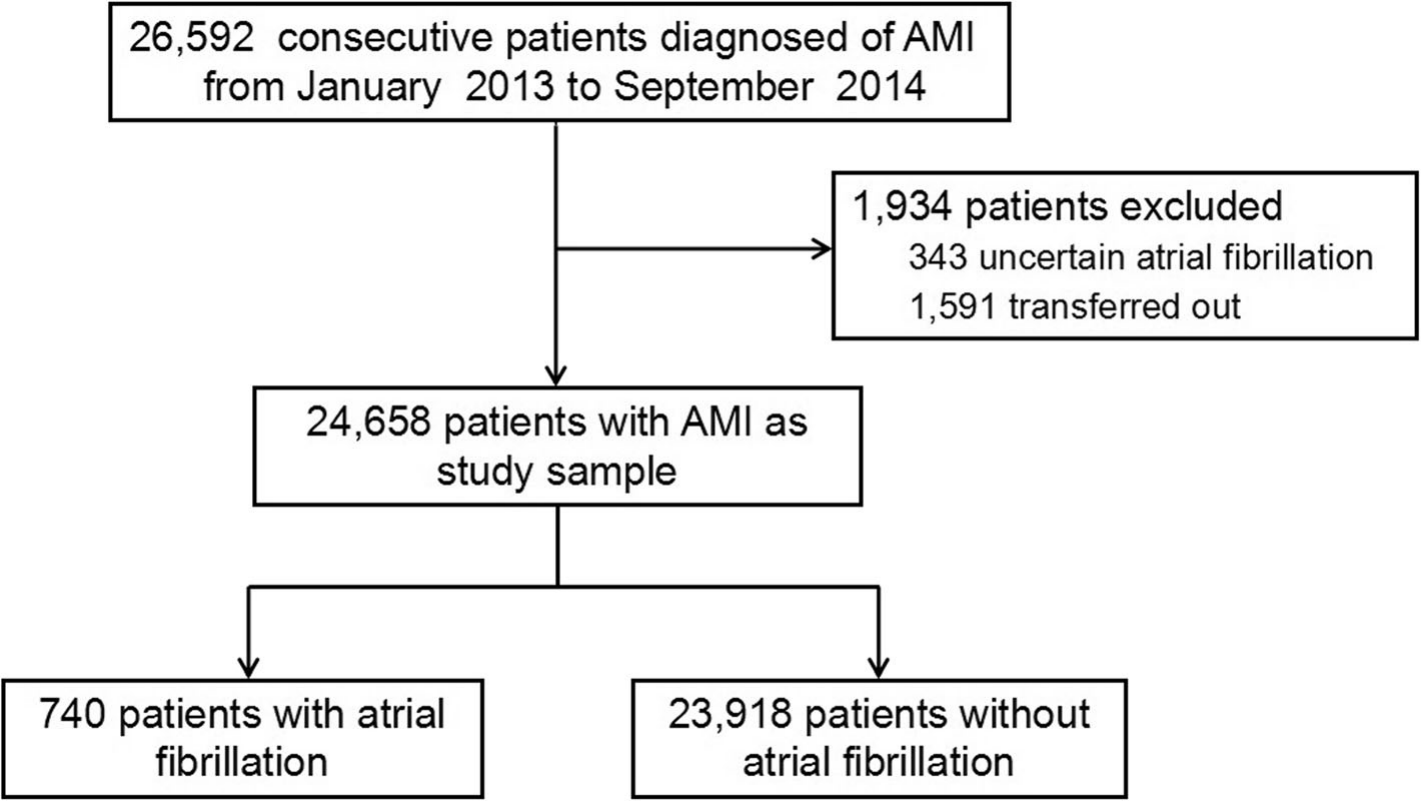
Population flow chart. AMI = acute myocardial infarction

Twenty-six thousand five hundred ninety-two patients diagnosed with AMI were consecutively enrolled in CAMI registry from January 2013 to September 2014. After excluding 343 patients with uncertain AF status and 1,591 patients who were transferred out during hospitalization, 24,658 patients were finally included in this analysis. Among them, 740 (3.0%) patients were recorded with AF prevalence during hospitalization (Fig. 1).

Baseline characteristics of patients were shown (Table 1). Compared with patients without AF, the age of patients with AF were higher (mean age: 73 vs. 63 years, *p* <0.01), more likely to be women (35.1% vs. 25.5%, *p* <0.01) and with more comorbidities such as hypertension (59.3% vs. 51.2%, *p* <0.01), heart failure (7.7% vs. 2.4%, *p* < 0.01) and stroke (17.8% vs. 9.2%, *p* < 0.01).Patients with AF were less frequently presented with STEMI than those without AF (65.7% vs. 76.0%, *p* <0.01), and had worse left ventricular function. The proportions of CHA2DS2-VASc ≥2 (66.1% vs.45.5%, *p* <0.01) and HAS-BLED ≥3 scores (21.4% vs.10.8%, *p* <0.01) were significantly higher in patients with AF, as well as the GRACE (161 vs.129,*p* <0.01) and Thrombolysis in Myocardial Infarction (TIMI) scores (6 vs.4,*p* <0.01). Patients with AF received reperfusion/revascularization during hospitalization at a lower rate than those without AF (35.9% vs. 48.3%, respectively, *p* < 0.01), as the case for PCI(29.7% vs. 40.5%, respectively, *p* <0.01).

**Table 1:** Baseline characteristics

	Overall population (*n* = 24658)	AF (*n* = 740)	No AF (*n* = 23918)	*P* value
Demographics				
Age(years)	63 (53–72)	73 (65–79)	63 (53–72)	<0.01
Men	74.2	64.9	74.5	<0.01
Medical history				
Previous angina pectoris	28.2	32.0	28.0	0.02
PreviousMI	7.7	7.9	7.6	0.81
PreviousPCI/CABG	5.2	4.5	5.2	0.37
Previous heart failure	2.6	7.7	2.4	<0.01
Previous stroke	9.5	17.8	9.2	<0.01
Previous peripheral arterial disease	0.6	1.1	0.6	0.15
Chronic renal failure	1.4	1.7	1.4	0.60
Cardiovascular risk factors				
Hypertension	51.5	59.3	51.2	<0.01
Hyperlipidemia	8.0	6.3	8.1	0.10
Diabetes mellitus	20.0	18.8	20.0	0.44
Family history of premature CAD	4.0	3.0	4.0	0.12
Current smoker	54.7	44.7	55.0	<0.01
Clinical characteristics				
STEMI	75.7	65.7	76.0	<0.01
LVEF(%)	55(47–60)	50(41–59)	55(47–60)	<0.01
Killip classification III-IV	9.2	24.2	8.7	<0.01
CHA2DS2-VASc ≥ 2	46.1	66.1	45.5	<0.01
HAS-BLED ≥ 3	11.1	21.4	10.8	<0.01
GRACE Score	129 (112–149)	161 (133–182)	129 (112–146)	<0.01
TIMI Score				
STEMI	4 (2–6)	6 (4–8.5)	4 (2–6)	<0.01
NSTEMI	2(2–3)	2(2–3)	2(1–3)	<0.01
Treatments				
Reperfusion Therapy	48.0	35.9	48.3	<0.01
PCI	40.2	29.7	40.5	<0.01
Fibrinolysis	7.8	6.2	7.8	<0.01
ACE/ARB	59.7	54.8	59.9	0.02
β-blockers	69.9	59.9	70.2	<0.01
Anti-arrhythmia drugs	9.8	45.4	8,7	<0.001

Data are presented as median (IQR) or %

*AF* atrial fibrillation, *MI* myocardial infarction, *PCI* percutaneous coronary intervention, *CABG* coronary artery bypass graft, *CAD* coronary artery disease, *LVEF* left ventricular ejection fraction, *GRACE* global registry of acute coronary events, *TIMI* thrombolysis in myocardial infarction, *STEMI* ST-segment elevation myocardial infarction, *NSTEMI* non-ST-elevation myocardial infarction, *ACEI* angiotensin-converting enzyme, *ARB* angiotensin receptor blocker

The antithrombotic treatment regimens in AMI patients with and without AF were summarized (Table 2). During hospitalization, 78.0% of patients with AF received DAPT, less than the rate of 86.3% in patients without AF (*p* <0.01). However, the rates of anticoagulants treatment including unfractionated heparin (UFH), low molecular weight heparin (LMWH) and fondaparinux both groups were similar. A majority of patients received DAPT (86.1%) and LMWH (84.2%). Only 3.5% of patients with AF received warfarin, which was nonetheless higher than the rate of 1.4% in patients without AF (*p* <0.01).

**Table 2:** Antithrombotic treatment strategy in-hospital and at hospital discharge

	Overall population (*n* = 24658)	AF (*n* = 740)	No AF (*n* = 23918)	*P* value
In-hospital^a^				
DAPT	86.1	78.0	86.3	<0.01
UFH	5.9	5.2	5.9	0.43
LMWH	84.2	84.9	84.1	0.59
Fondaparinux	3.8	2.9	3.9	0.16
Warfarin	1.4	3.5	1.4	<0.01
At hospital discharge				
DATP	85.9	76.2	86.1	<0.01
Warfarin	1.9	5.1	1.4	<0.01
Warfarin alone	0.3	1.0	0.3	0.02
Warfarin + single antiplatelet drug	0.9	2.4	0.7	<0.01
Warfarin + DAPT	0.7	1.7	0.5	<0.01

*AF* atrial fibrillation, *DAPT* dual antiplatelet therapy, *UFH* unfractionated heparin, *LMWH* low molecular weight heparin

^a^Not including anticoagulants administered in catheterization laboratory

At hospital discharge, 76.2% of patients with AF received DAPT, which was lower than the rate of 86.1% in patients without AF (*p* <0.01). However, only 5.1% of patients with AF were discharged on warfarin, and the proportion of warfarin in combination with DAPT was as low as 1.7%. In addition, no new direct oral anticoagulants (dabigatran, rivaroxaban, and apixaban) were applied in any patient.

**Fig. 2:**
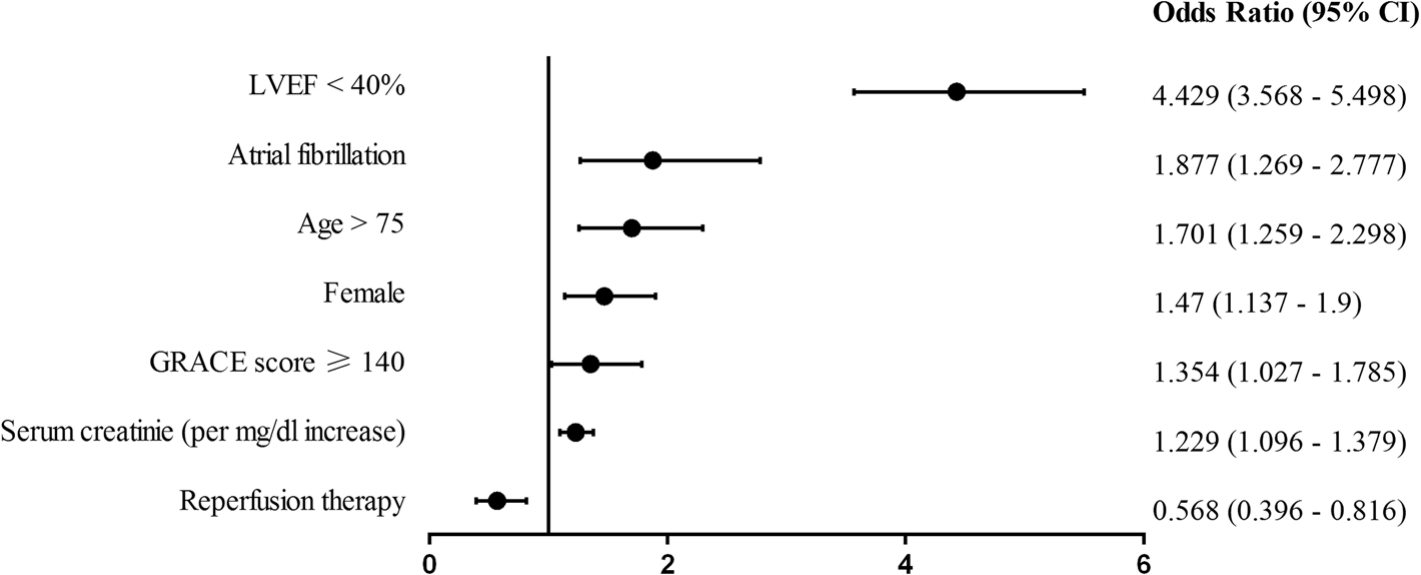
Multivariable analysis of predictors of in-hospital mortality*. * In-hospital mortality included in-hospital death and treatment withdrawal. LVEF = left ventricular ejection fraction; GRACE = Global Registry of Acute Coronary Events

**Fig. 3:**
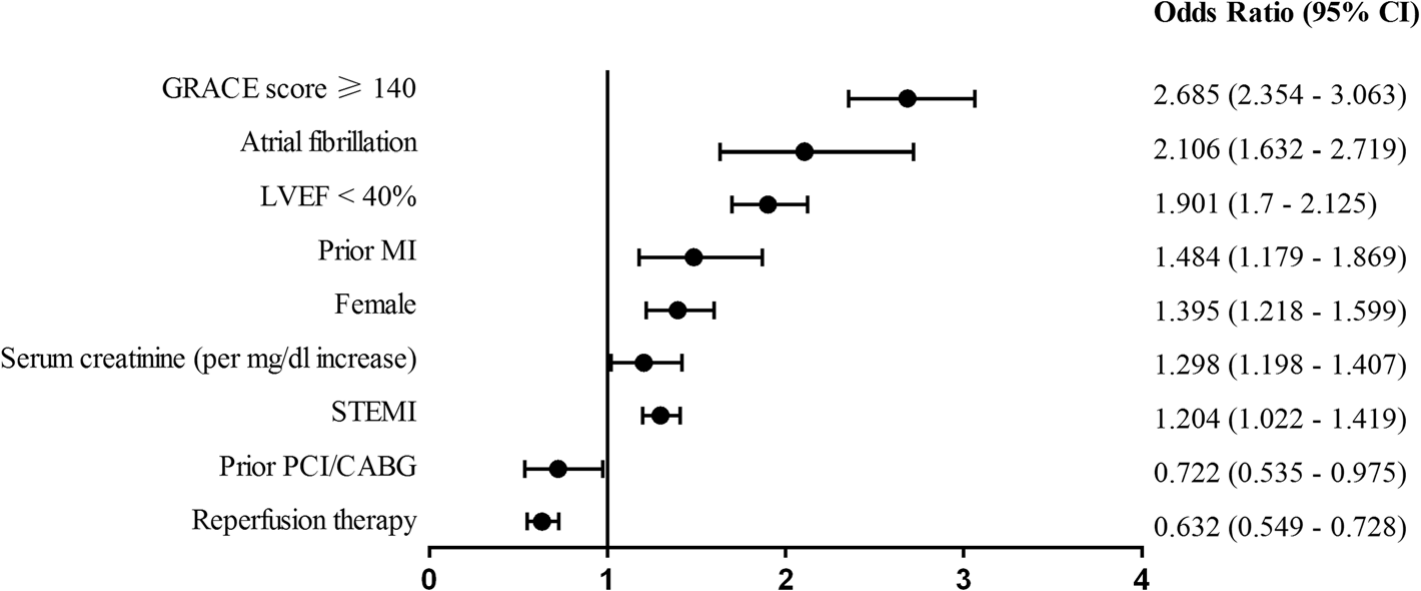
Multivariable analysis of predictors of the composite of adverse events*. *The composite of adverse events included in-hospital death, treatment withdrawal, re-infarction, heart failure or stroke. GRACE = Global Registry of Acute Coronary Events; LVEF = left ventricular ejection fraction; MI = myocardial infarction; STEMI = ST-segment elevation myocardial infarction; PCI = percutaneous coronary intervention; CABG = coronary artery bypass graft

The in-hospital outcomes were summarized (Table 3). Rate of in-hospital mortality (death or treatment withdrawal) was significantly higher in patients with AF (25.2%) than that of without AF (7.2%) (*p* <0.01). The rate of composite of adverse events (death, treatment withdrawal, re-infarction, heart failure or stroke) was also significantly higher in AF group (42.1% vs. 16.0%, *p* <0.01), which was also the case for individual component of the composite. In multivariate logistic regression analysis, AF was an independent predictor for both in-hospital mortality (odds ratio: 1.88, 95%CI: 1.27–2.78) and the composite of adverse events (2.11, 95% CI: 1.63–2.72, respectively) (Figs. 2 and 3). The rate of major bleeding was 1.7% in patients with AF, numerically higher than the rate of 0.9% for patients without AF (*p* =0.65).

**Table 3:** In-hospital events

	Overall population (*n* = 24658)	AF (*n* = 740)	No AF (*n* = 23918)	*P* value
Death	4.4	14.0	4.1	<0.01
Treatment withdrawal	3.3	11.2	3.1	<0.01
Death + Treatment withdrawal	7.7	25.2	7.2	<0.01
Re-infarction	0.6	1.4	0.6	0.02
Stroke	0.8	1.9	0.7	<0.01
Ischemic	0.6	1.4	0.5	
Hemorrhagic	0.08	0.13	0.07	
Unknown	0.11	0.4	0.09	
Heart failure	16.7	42.1	16.0	<0.01
Composite of adverse events^a^	19.2	47.5	18.4	<0.01
Major bleeding^b^	0.9	1.7	0.9	0.65
Any bleeding	1.8	2.7	1.8	0.09

^a^Composite of adverse events: death, treatment withdrawal, re-infarction, heart failure or stroke

^b^Major bleeding was defined as an absolute hemoglobin decrease of 3 g/dL, intracranial hemorrhage, any red blood cell transfusion or a bleeding event requiring surgical repair

## Discussion

CAMI registry was the largest nationwide observational study to date for hospitalized patients with AMI throughout China. The major findings of present analysis were: 1) the overall incidence of AF was 3.0% in Chinese patients with AMI during hospitalization; 2) the risk of baseline profile was higher in patients with AF than patients without AF; 3) patients who developed AF were at a 1.88-fold higher risk of in-hospital mortality than patients without AF; and 4) although the majority of AMI patients complicated with AF received anticoagulation and antiplatelet therapy during hospitalization, only 5.1% of them were discharged on warfarin, and 1.7% were discharged on both warfarin and DAPT.

In this nationally representative study, it firstly defined an AF incidence of 3.0% in contemporarily treated AMI patients in China. It was much lower compared to the reported data in other countries, ranging from 2.3 to 21% [1–12]. It may be resulted from some possible explanations. First, age was the most commonly reported risk factor for AMI complicated with AF [21, 22], and the low rate of AF in CAMI patients may be associated with an overall lower mean age of 63 years in samples. Second, 48.0% of overall patients in CAMI received reperfusion therapy (42.2% PCI). In previous studies, widespread use of reperfusion therapy, especially PCI, was associated with a notable decline of AF incidence [11, 23]. Third, the majority of patients in CAMI were treated with angiotensin-converting enzymes/angiotensin receptor inhibitors or β-blockers, and trials evaluating the effects of these drugs in patients with AMI have reported lower incidence rates of AF, although mainly making effects on late developing AF [24, 25]. Fourth, ethnic differences may also account for the wide incidence range of AMI complicated AF among different countries. A recently published study reported a low AF incidence of 2.7% in Arabian Gulf patients with acute coronary syndrome (ACS) [4].

Consistent with previous studies [1–12], in CAMI registry, higher-risk baseline clinical characteristics could be observed in AMI patients complicated with AF during hospitalization, including older age, a greater cardiovascular risk factor burden, more comorbidities, poorer left ventricular function, and higher clinical risk scores. The present study also documented that AMI patients with AF were less likely to receive reperfusion/revascularization than those without AF. For the patients with older age and more comorbidities, more conservative management approach would be selected by the physicians [26].

It indicated that AF increased the risk of morbidity and mortality in patients with ACS, and that this association would be mediated to a greater or lesser extent by various comorbidities [1]. However, because of differences in study design and data availability, including study population, AF classification, sample size, and follow-up duration, the association between AF development in ACS and increased in-hospital mortality remained to be controversial. Some variables were reported to be independently associated with AF [2–9], while others reported no association [10–12]. In present analysis, the data was obtained from the CAMI registry, which was a large-scale, national and contemporary registry project for AMI patients in China [20]. The in-hospital mortality was significantly higher in patients with AF in unadjusted analysis. In addition, AF was also an independent multivariate risk factor of mortality after adjusting for possible confounders, although to an attenuated extent. With the consistency of findings, the association was further underscored in unadjusted and adjusted analyses.

The risk of bleeding may be increased by the anticoagulants treatment combined with DAPT therapy for stroke prevention in ACS patients with AF [27, 28]. However, current guidelines and consensus recommend a combination of warfarin and DAPT (triple therapy), with adjustment of duration according to hemorrhagic risk [13, 14]. Nonetheless, in previous studies, it documented that this triple therapy was largely underused, with a frequency ranged from 5.7 to 15.6% [15–17]. In the CAMI national registry, only 5.1% of AMI patients with AF were discharged on warfarin, and the proportion of warfarin in combination with DAPT was even as low as 1.7%. The latter striking gap in China might be secondary to many factors: the uncertainty about the benefits of intense anticoagulation in these high risk patients, inadequate provider knowledge, structural inadequacies of healthcare delivery systems, and/or concern about potential violence and litigation from patients or their families due to complications associated with treatment [29–31]. In addition, although new direct oral anticoagulants (dabigatran, rivaroxaban, and apixaban) have been approved for stroke prevention in non-valvular AF patients [13], the CAMI registry indicated that these new anticoagulants have not been applied yet in AMI patients with AF in China.

CAMI registry was compared with REAL (REgistro regionale AngiopLastiche dell’Emilia-Romagna) registry. REAL registry was a multi-center, large scale, prospective study [32–35]. It aimed to collect the clinical data of coronary interventional cases from 4 million residents in Emilia- Romagna. 13 hospitals participated in this registry. The data could be retrieved in database. Many studies were performed based on this database [36]. Similar to REAL registry, CAMI has collected information of patients with acute myocardial infarction (AMI), including the clinical data, treatment, efficacy and prognosis. It aimed to improve the overall treatment efficacy of AMI in China. However, CAMI has only focused the patients from China. Different from REAL registry, CAMI has involved 108 hospitals in Chinese mainland and the hospitals differed in levels in CAMI registry. In addition, the population base was larger in CAMI registry in China. Finally, the involved cases were updated (since 2013). The study based on CAMI would be promising in improving the treatment efficacy of AMI in China.

### Strengths and limitations

CAMI is the largest national registry of patients with AMI. The population in the registry was representative of different regions, economic strata and access to medical resources in China. Therefore, the CAMI registry can adequately reflect the current performance and status of healthcare system in China. The data was valuable, specific and updated, which was based on a larger population base. Nevertheless, our study has several limitations. First, CAMI was subject to inherent limitations and potential biases, including the collection of nonrandomized data, missing or incomplete information, and potential confounding by drug indications or other unmeasured covariates which must be considered in results interpretation. Second, our database did not allow the identification of timing, type and duration of AF (paroxysmal, persistent or permanent), as well as the AF history, which may make effects on the prognosis prediction of the patients. Third, we do not include the follow-up data after hospital discharge, including both the mortality and other clinical events.

## Conclusions

In China, AF development in patients with AMI was associated with significantly higher in-hospital mortality, and the anticoagulants including warfarin were largely underused during hospitalization and after hospital discharge. The conclusion on prediction and treatment may be instructional towards both clinical practice and further relevant studies.

## Abbreviations

AF: Atrial fibrillation
AMI: Acute myocardial infarction
DAPT: Dual antiplatelet therapy
CAMI: China acute myocardial infarction
PCI: Percutaneous coronary intervention
CABG: Coronary artery bypass graft
STEMI: ST-segment elevation myocardial infarction
GRACE: Global registry of acute coronary events
OR: Odds ratio
CI: Confidence interval
TIMI: Thrombolysis in myocardial infarction
UFH: Unfractionated heparin
LMWH: Low molecular weight heparin
ACS: Acute coronary syndrome
